# The role of active learning methods in teaching pharmaceutical care – Scoping review

**DOI:** 10.1016/j.heliyon.2023.e13227

**Published:** 2023-01-26

**Authors:** Beata Plewka, Magdalena Waszyk-Nowaczyk, Magdalena Cerbin – Koczorowska, Tomasz Osmałek

**Affiliations:** a Pharmacy Practice Division, Chair and Department of Pharmaceutical Technology, Poznan University of Medical Sciences, 6 Grunwaldzka Street, 60-780 Poznan, Poland; b Department of Medical Education, Poznan University of Medical Sciences, 7 Rokietnicka Street, 60-806 Poznan, Poland; c Chair and Department of Pharmaceutical Technology, Poznan University of Medical Sciences, 6 Grunwaldzka Street, 60-780 Poznan, Poland

**Keywords:** Pharmacy students, Active learning methods, Pharmacy curriculum, PC, pharmaceutical care, CBL, case-based learning, PBL, problem-based learning, OSCE, Objective Structured Clinical Examination

## Abstract

**Background:**

The pharmacists in a community pharmacies have already provided, or will provide in the near future advanced pharmaceutical care services. This requires modifying the approach to teaching pharmacy students as well as adapting the curriculum to the changing professional realities. It has been proven that in the field of medical and related sciences, learners-centered active teaching methods allow to achieve learning outcomes effectively, especially in the field of practical skills.

**Objectives:**

As the pharmaceutical services are only being introduced in many European countries, the question arises as to what active learning methods to use to prepare pharmacy graduates for this. Thus the review of worldwide literature occurred to be helpful in identifying what active learning methods are being used specifically in teaching aspects of pharmaceutical care.

**Methods:**

Three electronic databases: Pubmed, Scopus and Web of Science were searched using the keywords “active learning” and “pharmaceutical care”.

**Results:**

On the basis of the publications included in the review, 7 methods were distinguished. Case-study, role play and simulation exercises turned out to be the most popular. It was also possible to make preliminary conclusions on how to properly match the method to the learning outcomes. Moreover, a weak point of many studies was the lack of structured methods of assessing the skills acquired by the students.

**Conclusions:**

In conclusion, the curriculum renewal in pharmacy is necessary and requires taking many aspects into account, from the types of tasks assigned to pharmacists, through the selection of appropriate teaching methods, to the verification of assessment methods.

## 1 Introduction

Both in Poland, under the Act on the profession of pharmacist, and in the European Union, under the resolution CM/Res (2020) 3 approved by the Council of Europe, the concept of pharmaceutical care is in force in accordance to the definition of Hepler and Strand. These authors defined pharmaceutical care as “the responsible provision of drug therapy for the purpose of achieving definite outcomes that improve a patient's quality of life” which “involves the process through which a pharmacist co-operates with a patient and other professionals in designing, implementing and monitoring a therapeutic plan that will produce specific therapeutic outcomes for the patient” [[1], [2], [3]]. On the basis of the above-mentioned documents, the concept of pharmaceutical services, such as a medication review, new medicine service, developing an individual care plan or carrying out diagnostic tests and vaccinations, was introduced in Poland [2,3]. This, in turn, requires not only the implementation of appropriate training in the process of continuing education of practicing pharmacists, but most of all modification of the curriculum during studies so that future graduates are substantially and practically prepared for the provision of pharmaceutical services. The need for changes in curricula has already been recognized in Europe and in the world. As a result more activities using active learning methods are gradually being introduced to them. One example is the United States, where the Accreditation Council for Pharmacy Education issued a recommendation that in the first three years of pharmaceutical studies, a minimum of 5% of all classes (300 h) should pose a program called Introductory Pharmacy Practice Experiences (IPPEs). IPPEs classes are designed to familiarize students with the practical aspects of a pharmacist's work in a real environment. The IPPE program prepares students for the Advanced Pharmacy Practice Experience (APPE) course, which takes place in the 4th year of studies with a duration of 1440 h. The APPE classes are designed to integrate and develop the knowledge, competences and attitudes developed during the IPPE [[4], [5], [6]]. On the other hand, in Europe, the curriculum at the Robert Gordon University in Aberdeen, Scotland, provides for the introduction of PC classes in the fourth year of studies, named: Pharmaceutical Care Provision and Specialized Pharmaceutical Care Provision. They are aimed at consolidating all previously acquired skills and knowledge in the pharmaceutical care process [[7], [8], [9]]. This is in line with World Health Organization (WHO) guidelines, which assume that curricula in medical faculties, in addition to knowledge and skills that are specific to the category of healthcare providers, should include competencies that will enable graduates to use them in the future correctly. Thus the medical educators are to provide practical experiences for their learners, necessary to achieve competencies in the fields of interpersonal communication, decision-making and management [10].

To ensure the effectiveness of the learning process the importance of a learner's involvement has been emphasized for many years, especially in such concepts as andragogy, experiental learning or deep learning, to list a few [[11], [12], [13], [14]]. Learners-centered, active learning methods allow to achieve learning outcomes effectively, mostly in the field of practical skills, so important in healthcare professions [[15], [16], [17]]. There are many active learning strategies among which clinical education, evidence-based medicine exercises and medical-based simulations are specific to medical sciences [18].

In view of the successively increasing range of services provided by pharmacists in the world and the consequent need to constantly monitor and adapt the curricula at pharmaceutical faculties to the current health needs of societies, the aim of this work is to identify, on the basis of the literature, the possibility of using active learning methods in teaching pharmaceutical care.

## 2 Methods

The scoping review was conducted as the purpose of the study was clarify concepts and identify knowledge gaps regarding active learning methods used in teaching pharmaceutical care at pharmacy faculties. The objective of the review is in accordance to the definition of scoping review by Munn et al. [19]. The intention of the authors was to map the issue and identify the concepts of student-centered teaching methods in hands-on PC teaching, rather than recommending specific learning solutions.

**Fig. 1:**
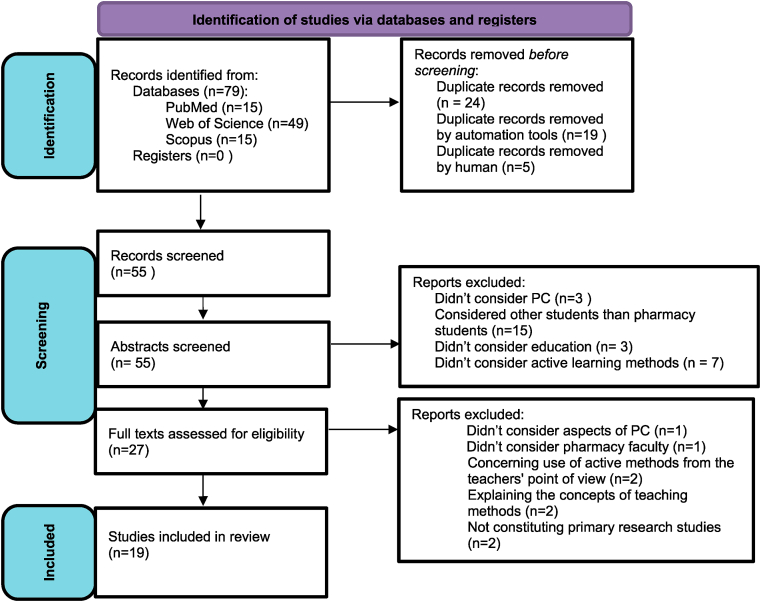
Review of the literature with PRISMA 2020 flow diagram. *Adapted from:* Page MJ, McKenzie JE, Bossuyt PM, Boutron I, Hoffmann TC, Mulrow CD et al. The PRISMA 2020 statement: an updated guideline for reporting systematic reviews. BMJ 2021; 372:n71. https://doi.org/10.1136/bmj.n71.

The authors searched 3 electronic databases: Pubmed, Scopus and Web of Science using keywords “active learning” and “pharmaceutical care” as they occurred to yield the most specific outcomes. Initial searches with other combinations of words provided too many results not associated with future pharmacists. Also using more detailed phrases brought no outcomes. The 2011–2022 date range was selected and keywords were searched under title/abstract or topic for the most relevant publications. The narrowing down of the scope of the years was aimed at obtaining the results that would best correspond to the current realities due to the dynamically changing role of the pharmacist in recent years and thus the necessity to update the curricula. PRISMA for Scoping Reviews guidelines were used in the preparation of the review and the PRISMA flow diagram adapted for the purposes of this study was utilized [20,21] (Fig. 1).

Seventy-nine results were obtained and imported into EndNote by Clarivate's citation manager, online version, to eliminate duplicate entries. Then the list was assessed by the researchers to check if every duplicated item was deleted. In the next step the researches screened abstracts of included records. At this stage, primary research studies that concerned teaching pharmaceutical care at pharmacy faculties using any active learning method were included in the review. On this basis, 28 publications which did not concern education, did not consider the use of active learning methods, or which did not focus on teaching pharmaceutical care or were related to students other than from pharmacy faculties, were excluded. Finally 27 items were screened to assess full-text in terms of eligibility for the review. Eight of them didn't meet the inclusion criteria, which were the same as for the abstracts. Two reports didn't concern aspects of running PC or pharmacy faculty and two focused on examining medical teachers' beliefs about active learning methods. Two aimed to clarify the concepts of teaching and assessing methods, including active learning methods, that can be used in the education of future pharmacists. Another two were not original articles. Each of the 19 included for review publications was analyzed in terms of the type of teaching method used and the aspect of pharmaceutical care it related to. If such data were available, the assessment tests and attitudes of students towards the method used would be also taken into account.

## 3 Results

The publications included in the review are presented in Table 1. The analysis of the manuscripts included obtaining information on what aspect of a pharmaceutical care was covered by the classes conducted by the authors of the study, what method of an active learning was used and what the results of the assessment of the introduced educational intervention were.

**Table 1:** List of publications qualified to the review, including the year of publication, place, aspect of the pharmaceutical care (PC), the educational methods and the evaluation methods used.

Author (year of publication)	Place (name of school or town/country)/level of studies	Aspect of pharmaceutical care	Active learning method	Evaluation
Benedict & Schonder (2011) [22]	University of Pittsburgh School of Pharmacy/third-year doctor of pharmacy (PharmD)	pharmaceutical care to critically ill patients and patients with kidney disease	PharmaCAL – a software to create patients simulation. Students were given challenges with options to choose a procedure; each choice was followed by feedback about specific consequences.	Students found software as useful and satisfactory tool. The pre/post assessment of knowledge revealed improvement in knowledge (p < 0.0001)
Woelfel et al. (2011) [23]	The School of Pharmacy at the Thomas J. Long School of Pharmacy and Health Sciences; USA/Introductory Pharmacy Practice Experiences (IPPEs); first and second year.	Geriatric care	Visit at geriatric-care facility. Students' tasks: - Interviews with patients and caregivers and reviewing medical records - identifying the patient's problems and creating a care plan	The course was rated consistently higher than other School of Pharmacy courses (p = 0.027).
Campbell et al. [24]	McWhorter School of Pharmacy, Samford University, Birmingham, AL/first-year students	PC on patients with myocardial infarctions	Case-based activity in which students were presented a real-life patient scenario in which the task was to conduct an interview, verify pharmacotherapy and provide information about drugs	Improve in knowledge: the post-test result was significantly higher than that of the pre-test (p < 0.01) Students agreed the exercise met the assumptions, but had a moderate impact on drug literature knowledge and searching skills
Sterrett et al. (2012) [25]	South Carolina College of Pharmacy/third-year doctor of pharmacy (PharmD)	Diabetes care	1. instructor-led modeling and role-playing exercises, 2. small group activities and objective structured learning exercises (OSLE) using standardized patients on interviewing patient and practical skills: blood pressure and glucose monitoring, foot examination, 3. simulation	Students, perceived confidence in diabetes care - ranged from 4.2 to 4.8 on 5-point Likert scale Students' satisfaction – 4.5 Students' perceived usefulness of the course in practice – 4.8
Katoue & Al Haqan (2013) [26]	Faculty of Pharmacy at Kuwait University, Kuwait/final-year pharmacy students	patient counseling about antidiabetic medications	Case study discussions in small groups and role-play in pairs	All students (100%) strongly agreed that the workshop was very useful. Case study discussion in small groups was rated as the most preferred learning method by students. Scores in post-test of knowledge was significantly higher than in pre-test (p < 0.05).
Limberger (2013) [27]	Franciscan University Center (UNIFRA)/the seventh semester of pharmacy	Identifying the medicines of choice; identifying and solving drug-related problems	Case study	Not available
Lucas et al. (2013) [28]	University of Charleston School of Pharmacy, Charleston, West Virginia/second year and third year of a doctor of pharmacy (PharmD) program	Pharmacotherapy: ” develop strategies to increase patient understanding, motivation, and adherence to treatment plans; identify and prevent drug-related problems; effectively monitor, assess, and optimize therapeutic plans; and apply current practice guidelines to therapeutic recommendations”	Discussion – based activities, e.g. patient cases	Subjective increase in the students' sense of responsibility and capability for the self-learning process
Norose (2013) [29]	Hokkaido Pharmaceutical University School of Pharmacy, Otaru, Hokkaido, Japan/fifth year of pharmacy	Creating care plan for patients according to SOAP – plan: Subjective information, Objective information, Assessment, and Plan	Problem – based learning using SP (standardized patients) method	Students' comments: the experience helped to understand how to create a care plan and the importance of interview with the patient in that process
Suno et al. (2013) [30]	Medical Education Center, Okayama University/undergraduates	pharmaceutical care practices	Team – based learning	87.3 ± 9.3% students were satisfied with TBL method; individual readiness assessment test (IRAT) score before the TBL sessions were lower than the group readiness assessment test (GRAT) score during TBL
Waitzman & Dinkins (2013) [31]	UNC Eshelman School of Pharmacy, University of North Carolina, Chapel Hill, North Carolina/second-year pharmacy students	prescription analysis exercise – identification of errors and omissions, followed by their correction	Hands-on exercises with index card box with stock bottle cards, prescriptions and labeled medicine bottles	94% of students found the new method of teaching prescription validation more realistic and practical than the one used in previous semesters
Mesquita et al. (2015) [32]	Department of Pharmacy, Federal University of Sergipe, São Cristovão, Sergipe, Brazil/fourth year of the undergraduate pharmacy program	Philosophy of pharmaceutical care as well as core competencies, e.g. identification and management of patients' drug related needs	Dialogic classroom expository, role play, simulated patient, lecture, case studies, virtual patient	Students were examined with four methods: discursive written exam, seminars, OSCE, virtual patient. Mean score of all was 7.97 (±0.59) on a scale of 0–10 points. In pre/post test on self-assessment of pharmaceutical care skills a statistically significant (p < 0.05) increase in scores was obtained for all competences on the Likert scale. Over 90% of students were satisfied with the classes in terms of the teacher's work, feedback and usefulness of the course for professional/personal development, the possibility of acquiring knowledge and the form of classes itself
Gossenheimer et al. (2017) [33]	Federal University of Rio Grande do Sul, School of Pharmacy, Porto Alegre, Rio Grande do Sul, Brazil/undergraduates	PC in terms of legislation, drug dispensing, treatment adherence, medication related errors	Games, discussion, case – studies, development of a drug dispensing roadmap and conceptual map on treatment adherence, service and patient care simulation	The study aimed to compare distance education to face-to-face education using active learning methods. In terms of pharmaceutical services, students obtained higher grades in the e-learning format (p < 0.027). In terms of information about the drug, there were no statistically significant differences in the final ratings.
Martinez et al. (2017) [34]	Universidad Complutense de Madrid (SPAIN)/third-year pharmacy students	Clinical case of multiple sclerosis	Problem-based learning	Short-answer and essay exam after a week; the method enhanced communication skills, self-responsibility for learning and team cooperation in solving problems.
Czepula et al. (2018) [35]	Federal University of Parana, Brazil/undergraduates	Pharmaceutical Care (the authors did not specify the thematic scope)	Blended learning	Pre-test on the first day of classes and post-test at the end of semester; both were the same and built to test students according to 3 domains of Bloom's taxonomy. There was a statistically significant increase in the results in the post-tests compared to the pre-tests at each level of Bloom's taxonomy, in each thematic module.
Marvanova & Henkel (2018) [36]	Department of Pharmacy Practice, Chicago State University College of Pharmacy/third-year PharmD students	PC in Parkinson's disease and Alzheimer's disease	Short-case scenarios and hands-on activities, e.g. discussion, case-based scenarios	Pre/post tests Conducted before and after short-case scenarios revealed improvement in assessed domains: therapy recommendation and rationale (p < 0.05 to p < 0.01). Students' self-rated confidence assessed on Likert scale also increased significantly (p < 0.01), while interest in topic remained the same (p = 0.066). 85.6% stated that their knowledge improved “somewhat” or “very”.
Prescott & Nobel (2019) [37]	University at Buffalo School of Pharmacy and Pharmaceutical Sciences, Buffalo, New York/first year pharmacy students	Cultural competency	Global Bead and Trading Places; structured discussion; counseling sessions	In-class quiz: mean grade 86.1% Counseling score: mean grade 92.6% In students' perception, the trading places exercise was scored the lowest
Foppa et al. (2021) [38]	Department of Pharmaceutical Sciences, Health and Science Center, UFSC, Florianópolis, Brazil/undergraduates	Providing patient education, dispensing medicines, evaluation of the effectiveness and safety of pharmacotherapy	Teaching and Learning of Pharmacy Services (TLPS method) based on two components: a theoretical-reflexive (preparing protocols) and a practical-reflexive (using protocols in contact with the real patient)	Students were assessed retrospectively by teachers and supervisors based on specific criteria. The authors did not provide the evaluation results. Students reported improvement in communication, anamnesis and decision making.
Nasser et al. (2021) [39]	Lebanese American University, School of Pharmacy/second year pharmacy students	Pharmacists' Patient Care Process (PPCP): implementation and control of prescriptions, identification of drug problems, patient education, professional counseling, communication with representatives of other medical professions, preparation and presentation of protocols from the course of patient consultations	Case studies, student presentations, role play and minutes writes	A survey on the perception of the degree of preparation for running the Pharmacists' Patient Care Process was given to students before and after class and after completing the entire course in the next academic year. It was also filled in by the control group. The results showed an improvement in student readiness to validate prescriptions, interview the patient, and develop a pharmaceutical care plan that was sustained through another year of study.
Faustino et al. (2022) [40]	Faculty of Pharmaceutical Sciences, University of Sao Paulo/first year pharmacy students	Pharmaceutical services such as medicine selection and health education	creating virtual pharmaceutical games by students	In the opinion of students and teachers, the method was motivating and facilitating learning, although the students lacked the opportunity to play the games designed by individual groups.

### 3.1 Place and year of studies

Most of the authors conducted their research in the United States of America (n = 9) and in Brazil (n = 5). Two studies were carried out in Europe (Spain and England) and three in Asia (Japan and Kuwait). In four cases, the described classes were conducted as part of doctoral studies, all of them in the USA. Students of the first year of pharmacy also took part in the study 4 times, mainly in the USA and once in Brazil. In the remaining cases, they were undergraduate students of further years or the authors did not indicate the year of the studies.

### 3.2 Aspect of pharmaceutical care

Seven aspects of pharmaceutical care were identified and are presented in Table 2. Two manuscripts did not specify what form of pharmaceutical care the exercise concerned.

**Table 2:** The aspect of pharmaceutical care discussed in class.

PC aspect	Authors
Pharmaceutical care for a patient suffering from a specific disease (e.g. diabetes)	Benedict & Schonder (2011) [22] Campbell et al. (2012) [24] Sterrett et al. (2012) [25] Katoue & Al Haqan (2013) [26] Martinez et al. (2017) [34] Marvanova & Henkel (2018) [36]
Dispensing the prescription in the context of correctness, verification of medical recommendations and detection of drug problems	Limberger (2013) [27] Lucas et al. (2013) [28] Waitzman & Dinkins (2013) [31] Gossenheimer et al. (2017) [33] Nasser et al. (2021) [39]
Patient education (pharmacist as an educator)	Lucas et al. (2013) [28] Foppa et al. (2021) [38] Nasser et al. (2021) [39] Faustino et al. (2022) [40]
Patients' medicines related needs	Mesquita et al. (2015) [32] Faustino et al. (2022) [40]
Develop a pharmaceutical care plan	Woelfel et al. (2011) [23] Norose (2013) [29]
Geriatric care	Woelfel et al. (2011) [23]
Cultural competency	Prescott & Nobel (2019) [37]

### 3.3 Active learning methods

The simulation exercises were implemented in the case of 5 interventions (in USA, Japan and Brazil), 3 of them using the simulated patient method and 2 in a real environment in direct contact with patients. The most popular methods were the case study and role play, which were used by 6 and 4 researchers respectively. It turned out that virtual methods were equally eagerly used, both in distance and on-site learning. Four authors decided to use the active learning method in virtual reality. The methods and the authors of the studies are summarized in Table 3.

**Table 3:** Active learning methods used by the researchers.

Method	Definition	Authors
Case study	Case-based learning is a method that aims to prepare students to practice their profession through the use of authentic cases from community pharmacy setting. This allows to link theory with practice by using the substantive knowledge in solving the presented cases [41].	Katoue & Al Haqan (2013) [26] Limberger (2013) [27] Lucas et al. (2013) [28] Gossenheimer et al. (2017) [33] Marvanova & Henkel (2018) [36] Nasser et al. (2021) [39]
Role play	“Role play is a dramatic technique that encourages participants to improvise behaviors illustrating expected actions of persons involved in defined situations” [42]	Sterrett et al. (2012) [25] Katoue & Al Haqan (2013) [26] Mesquita et al. (2015) [32] Nasser et al. (2021) [39]
Simulation	High-fidelity experiences that use standardized patients or are conducted in a realistic conditions, e.g. in contact with the real patient [42]	Woelfel et al. (2011) [23] Sterrett et al. (2012) [25] Norose (2013) [29] Mesquita et al. (2015) [32] Foppa et al. (2021) [38]
Virtual exercises	“A computer generated display that allows or compels the user (or users) to have a sense of being present in an environment other than the one they are actually in, and to interact with that environment” [43]	Benedict & Schonder (2011) [22] Gossenheimer et al. (2017) [33] Czepula et al. (2018) [35] Faustino et al. (2022) [40]
Educational games	“Educational games (or serious games) are specifically designed to teach people about a certain subject, expand concepts, reinforce development, or assist them in drilling or learning a skill or seeking a change of attitude as they play” [44]	Prescott & Nobel (2019) [37]
Team-based learning	“The learner-centered, teacher-directed instructional approach for entire classes of students who are divided into small teams of between five and seven students to solve authentic problems” [45].	Suno et al. (2013) [30]
Problem-based learning	The student-centered approach in which small group of students, facilitated by a teacher are presented a real problem, which is the basis for identifying issues that need further self-study [45].	Campbell et al. (2012) [24] Martinez et al. (2017) [34]

### 3.4 Evaluation

Significant improvements were obtained in all studies that assessed student skills. Also in cases where students were asked to rate the classes, high satisfaction scores were obtained. Likert scale surveys were most often used to obtain students' opinions.

## 4 Discussion

In view of the changing role of pharmacists in community pharmacies, active learning methods can be particularly useful in providing pharmacy students with the necessary skills to deliver pharmaceutical services [46]. In order for the active learning methods used to bring the expected results, they should meet several conditions. Namely, it should be possible to provide feedback, the thematic scope must be integrated and complementary to the curriculum, and as an educational intervention, it is supposed to have defined and measurable learning outcomes and be used in an appropriate educational and professional context [47]. The results obtained in the analysis show that case-studies and various faces of simulation are particularly popular among active learning methods at pharmacy faculties and they meet the above mentioned conditions.

### 4.1 Case-study

Case-study method, also known as a case-based learning (CBL) is often compared with problem based learning (PBL) [48]. Although these are active learning methods in which students solve a specific problem which is the patient's case, as Williams points out, the main difference is that in the CBL method, students use the already acquired knowledge, while in the PBL method they identify areas that require self-study [49]. In a review, two authors: Campbell et al. and Martinez et al. used the PBL method, which was probably justified by the fact that the classes were attended by the first – and the third-year students who did not have adequate knowledge of the subject of the case, i.e. myocardial infarctions or multiple sclerosis [24,34]. Therefore, at this stage, the choice of this method seems to be adequate to the level of knowledge and students' skills, which is an important criterion in designing curricula [42,50]. In turn, Nasser et al. introduced changes to the module called Pharmacists' Patient Care Process in the second year of pharmacy, enriching it with active learning methods such as CBL and role play, and examined their impact on students' sense of readiness to provide services in five aspects of PC immediately after class and after one year after their completion [39]. Thanks to this, the authors proved that their classes increased students' self-confidence, which lasted for the next year of study in three aspects: prescription validation, interviewing the patient and creating a PC plan. Gossenheimer et al. [33], who also used the case study method, but in conjunction with virtual exercises, had a similar subjects of the classes in their study. Nonetheless, other authors used this method at more advanced stages of studies, including PharmD courses, in issues related to specific disease entities or the practical use of official guidelines [26,28,36]. In all studies that used the CBL method, students used the acquired knowledge and skills to solve the problem, which allowed them to combine the knowledge with the practice.

### 4.2 Simulation-based solutions

Pharmacists in Poland, and also on a global scale, are facing changes in their profession, which are a response to the growing health needs of societies [51]. In parallel, it is imperative that the pharmacy curriculum is refreshed to ensure an up-to-date, evidence-based curriculum that follows changes in practice. In the literature, this process is called curriculum renewal [4,50]. In pharmaceutical departments in recent years, this process also includes the introduction of active learning methods, especially simulation techniques [52,53]. Simulation as a method of teaching in medical faculties is the most advanced technique and usually associated with the use of part task trainer, full body simulator, screen simulator or virtual reality, real people as simulated patients as well as hybrid of those techniques [47]. However, if the simulation is considered in accordance to the definition of Gaba, i.e. as a technique, not technology, it can be defined as an educational method, which consists of confronting the student with real situations related to the future work and learning practical skills in safe and controlled conditions [[54], [55], [56]]. Thus, it is possible to expand the scope of simulation methods that can be used not only in teaching surgical and diagnostic skills, but also in any skill that requires contact with the patient. Simulation-based learning is therefore used in teaching future physicians, nurses, paramedics and, relatively shortly, pharmacists [[57], [58], [59], [60]]. As it can be assumed from this review, the simulation method is not as popular in teaching pharmacy students in Europe as in America. For example, in Poland, thanks to the support of the European Union, 12 Simulation Centers at Universities were established in 2015–2021, but the financing did not include the participation of pharmaceutical departments in the project [61]. Therefore, the costs of this type of classes may constitute an obstacle in their implementation in some European countries. On the other hand, simulation exercises may have a different level of advancement, i.e. a different level of realism. In this context, we can distinguish low fidelity and high fidelity simulations [42]. In teaching pharmacists, exercises with standardized patients or involving consultation with real patients is a high-fidelity simulation [52]. Nevertheless, there are authors that include case studies and role plays as well as virtual exercises in low fidelity simulations [42]. It is worth noting that in the studies qualified for the review, the authors often used case study methods and role play, which, although less advanced than high fidelity simulation, also brought the desired results. For example, in the Katoue and Al Haquan research, both methods were used, which translated into an improvement in the student knowledge test results and made 100% of the students rate the workshops as very useful [26]. Similar conclusions were reached by Marvanova and Henkel who, after introducing case studies on pharmaceutical care for patients with Parkinson's and Alzheimer's disease, noted an improvement in the results of knowledge and self-assessment of students' confidence [36]. Furthermore, the advantages of the high fidelity simulation classes were expressed by students in terms of better preparation for providing PC or other pharmaceutical services, but also a greater understanding of the importance of particular elements of PC, such as a correctly interviewed patient [23,29]. Moreover, the participants of the courses in which this method was used showed high satisfaction with the training and assessed its practical usefulness well [23,25,32]. Taking into account the assumptions of the Kirkpatrick model, according to which the participants' assessment of the level of satisfaction and usefulness of the content provided during a given course is the basis for its evaluation, these results confirm the high usefulness of all kind of simulation techniques [62]. Nevertheless, also in relation to the above-mentioned model, the proper method of checking the knowledge acquired by students shouldn't be forgotten. Meanwhile, only in a study by Mesquita et al. the OSCE exam was used as one of the methods of evaluating student achievement and thus the effectiveness of the educational method used [32]. While students often perceive this type of exam as stressful, both they and the teachers see many positive aspects, including the adequacy and fairness of the assessment [63]. For this reason, the results of such an exam are likely to be more objective than measuring the satisfaction with the course itself. It is interesting that in the study by Gossenheimer et al. in which online classes were compared with stationary classes, students obtained higher grades in tests on pharmaceutical services conducted using the e-learning method [33]. This may prompt in-depth research into virtual teaching methods.

### 4.3 Other issues

The aspect, related to the subject matter of the classes, that would be worth exploring, is the cultural competence of future pharmacists. This issue was discussed only in one study by Prescott & Nobel in which the game Global Bead and Trading Places was used [37]. This seems to be quite a serious gap in the teaching of future pharmacists, expressed by both students and practicing pharmacists [[64], [65], [66]]. Another issue is introducing an active learning methods in the early stages of pharmacists' education. Among the works included in the review, only four concerned the use of this type of method in the first year of pharmacy. The obstacle is probably the lack of theoretical foundations of the first-year students, but it seems that it would not be necessary in acquiring, for example, communication skills. On the other hand, research shows that pharmacists indicate that during their education they never had the opportunity to participate in an interpersonal communication classes [67]. It is also worth noting that communication skills can be taught independently of acquired professional knowledge and with the use of less advanced methods of active learning, such as debates or role-taking [68,69].

### 4.4 Limitations and prospects

The review of research on educational methods, in addition to providing information on the type and effectiveness of active learning methods, showed in our opinion areas that could be improved in this type of research. One aspect is the lack of a comparative group in the studies. It is highly probable that any educational intervention will result in learning. For this reason, the obtained assessments of a given intervention or changes in students' knowledge and attitudes, even obtained as a result of comparing pre- and post-studies, can be difficult to interpret. The solution could be to conduct a similar study among students who had the same issue discussed in class without the use of the active methods. However, we realize that it would not be acceptable in many cases to divide a group of students and teach some of them in a potentially less effective way. In such a situation, it might be helpful to carry out the same analysis of the effectiveness of a specific method after a certain period of time after the classes. This would allow to confirm the influence of active learning methods on knowledge retention and maintaining students' attitudes and contribute to accelerating the implementation of these methods in pharmaceutical curricula.

## 5 Conclusions

Active learning methods are used in teaching pharmaceutical care, but high-fidelity simulation exercises are not among the most commonly used methods, especially in Europe. This is probably due to financial constraints, which is why many more researchers decided to use the case study or role-play methods. Appropriate selection of educational methods to the learning outcomes conveyed in the pharmacy curriculum seems to be the way to resolve the situation. Simpler skills, such as communication skills, can be perfected using cheaper methods, and more complex ones, such as the pharmaceutical care of a chronically ill patient, using methods such as simulation exercises with the standardized patient. By far the weakest point was the evaluation of courses, which in most cases was limited to assessing student satisfaction, but lacked structured methods of assessing skills such as OSCE or objectified sense of students’ self-efficacy.

## Declarations

### Author contribution statement

All authors listed have significantly contributed to the development and the writing of this article.

### Funding statement

This work was supported by the National Science Center, Poland [Grant No. 2021/41/N/HS6/01359].

### Data availability statement

Data will be made available on request.

For the purpose of Open Access, the author has applied a CC-BY public copyright licence to any Author Accepted Manuscript (AAM) version arising from this submission.

### Declaration of interest’s statement

The authors declare no competing interests.
